# Early Postoperative Pulmonary Fat Embolism Following Cosmetic Liposuction: A Case Report

**DOI:** 10.7759/cureus.108164

**Published:** 2026-05-03

**Authors:** Rana A Almugassabi, Nikita S Jasani, Mariam S Alshehhi, Shama Y Lootah, Lamees Mohamedali, Leena Abdelrahman, Hina Zia Mirza

**Affiliations:** 1 General Medicine, Libyan International Medical University, Benghazi, LBY; 2 General Medicine, GMERS Medical College and Hospital, Sola, Ahmedabad, IND; 3 Internal Medicine, Mohammed Bin Rashid University of Medicine and Health Sciences (MBRU), Dubai, ARE; 4 Internal Medicine, University of Sharjah, Sharjah, ARE; 5 Internal Medicine, Dubai Hospital, Dubai, ARE

**Keywords:** anemia, deep vein thrombosis, fat embolism, fat macroglobulinemia, liposuction, pulmonary thromboembolism, respiratory distress, tachycardia, thrombocytopenia

## Abstract

Liposuction is a very common plastic surgery procedure. Post-liposuction complications are uncommon but can be life-threatening, and early cardio-pulmonary symptoms may mimic benign postoperative responses. Serious complications such as fat embolism syndrome (FES) and pulmonary embolism rarely present within the first few hours, and an atypically early onset can create significant diagnostic uncertainty. FES, in particular, may initially manifest with subtle or nonspecific symptoms, contributing to missed or delayed recognition.

We report the case of a 34-year-old previously healthy woman who presented to the emergency department with palpitations, dizziness, and hypotension approximately one hour after undergoing cosmetic liposuction under local anesthesia. Her presentation was atypical both in timing and in the absence of the classic triad of respiratory distress, neurological changes, and petechial rash. Given the unusually early onset, FES was considered but required careful differentiation from other postoperative causes of acute cardiopulmonary symptoms.

## Introduction

Liposuction is a commonly performed aesthetic procedure worldwide [1] that may be carried out using various techniques under local, regional, or general anaesthesia. Since its introduction in the 1970s, it has generally been regarded as a safe intervention, with low reported rates of major complications and an overall mortality estimated to be less than 0.02% [2].

With increasing procedural volume, there has been a proportional rise in reported complications. These range from minor adverse effects, such as seroma formation, contour irregularities, local anaesthetic toxicity, and hyperpigmentation, to severe and potentially fatal complications, including visceral injury, haemorrhage, venous thromboembolism, infection, necrotising fasciitis, and fat embolism [2]. Among these, fat embolism syndrome is one of the most serious complications associated with liposuction, with or without fat grafting, and is associated with significant morbidity and mortality [3]. 

Fat embolism syndrome represents a spectrum of clinical manifestations resulting from embolisation of fat particles into the systemic circulation. It has been most commonly described following long-bone fractures but is increasingly recognised in association with elective aesthetic procedures involving fat disruption or mobilisation. Although FES is relatively uncommon, it may progress rapidly and is associated with reported mortality rates ranging from 10% to 36% [3]. 

Diagnosis is challenging due to the absence of pathognomonic features and the nonspecific nature of laboratory and imaging findings, resulting in widely variable reported incidence rates, ranging from less than 1% to 30% [4]. FES typically develops within 12-72 hours following the inciting event and classically presents with pulmonary, neurological, and cutaneous manifestations. Pulmonary symptoms, such as dyspnea, tachypnea, and hypoxemia, are the most frequent and often the earliest features, occurring in approximately 75% of cases [5].

The treatment of FES is largely supportive, including respiratory and haemodynamic stabilisation, and prevention of complications [6, 7]. Corticosteroids may reduce mortality in severe FES, but their routine therapeutic use remains controversial [8]. Anticoagulation is not an established therapy for FES [8].

This case highlights the potential for fat embolism syndrome to present unusually early and without classical manifestations following cosmetic liposuction. 

## Case presentation

History and physical examination

A 34-year-old previously healthy woman presented to the emergency department with sudden-onset dizziness that worsened on standing, palpitations, diaphoresis, and numbness approximately one hour after undergoing elective abdominal liposuction under local anaesthesia. The procedure was performed minimally invasively via right and left three-millimeter inguinal incisions. Detailed operative records from the cosmetic clinic were unavailable. The patient denied chest pain, shortness of breath, syncope, nausea, vomiting, or loss of consciousness. She reported mild peri-umbilical abdominal discomfort and noted the appearance of a rash over both lower limbs.

**Figure 1:**
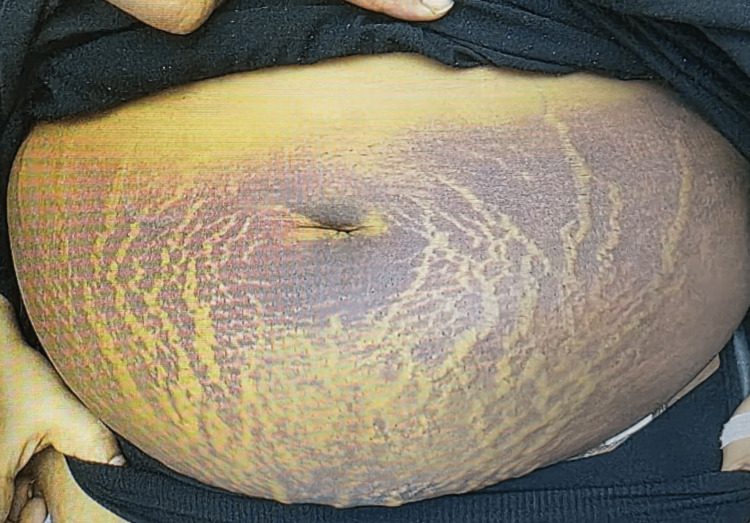
Chemosis following abdominal liposuction

On arrival, the patient was alert and oriented. Vital signs revealed marked sinus tachycardia with a heart rate of 160 beats per minute and hypotension with a blood pressure of 105/59 mmHg. Oxygen saturation remained within normal range on room air. Abdominal examination demonstrated diffuse tenderness, post-liposuction soft-tissue oedema, and ecchymosis (Figure 1).

**Figure 2:**
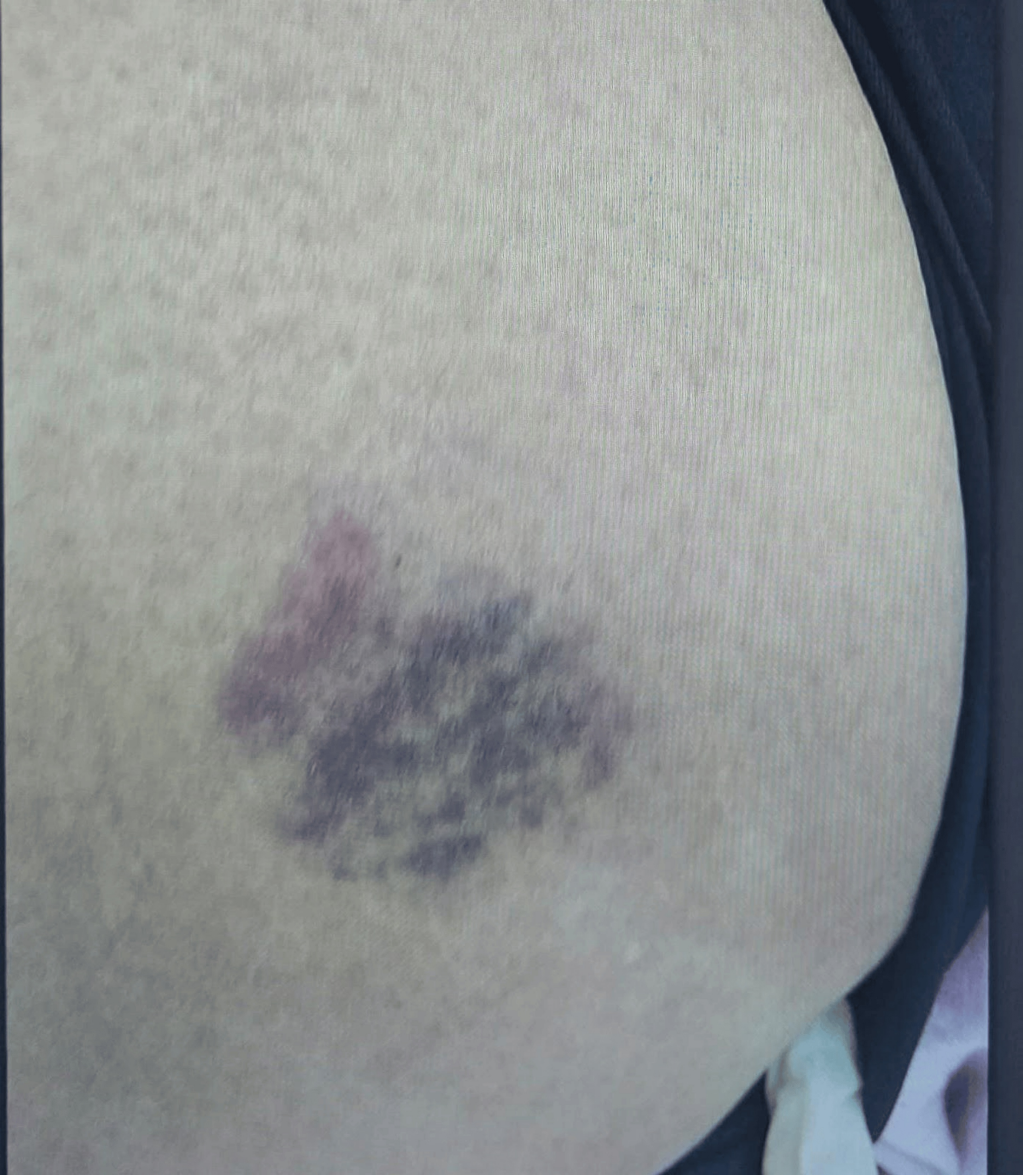
Petechial rash on lower left back

**Figure 3:**
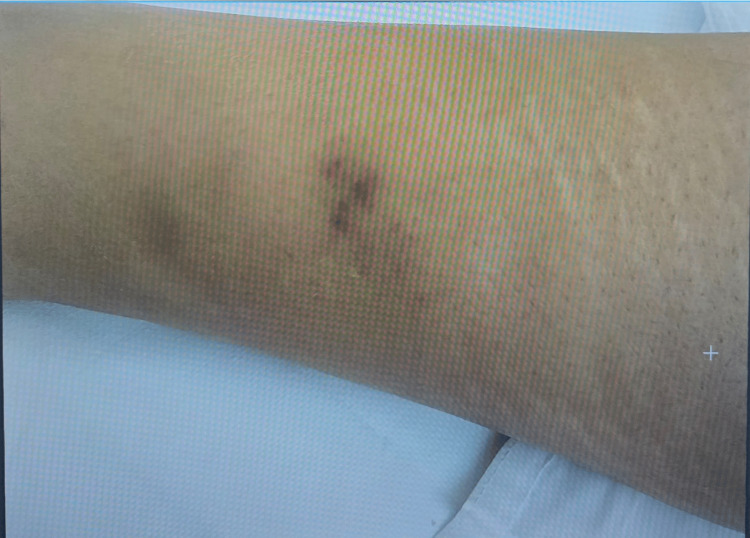
Petechial rash on right lower limb

**Figure 4:**
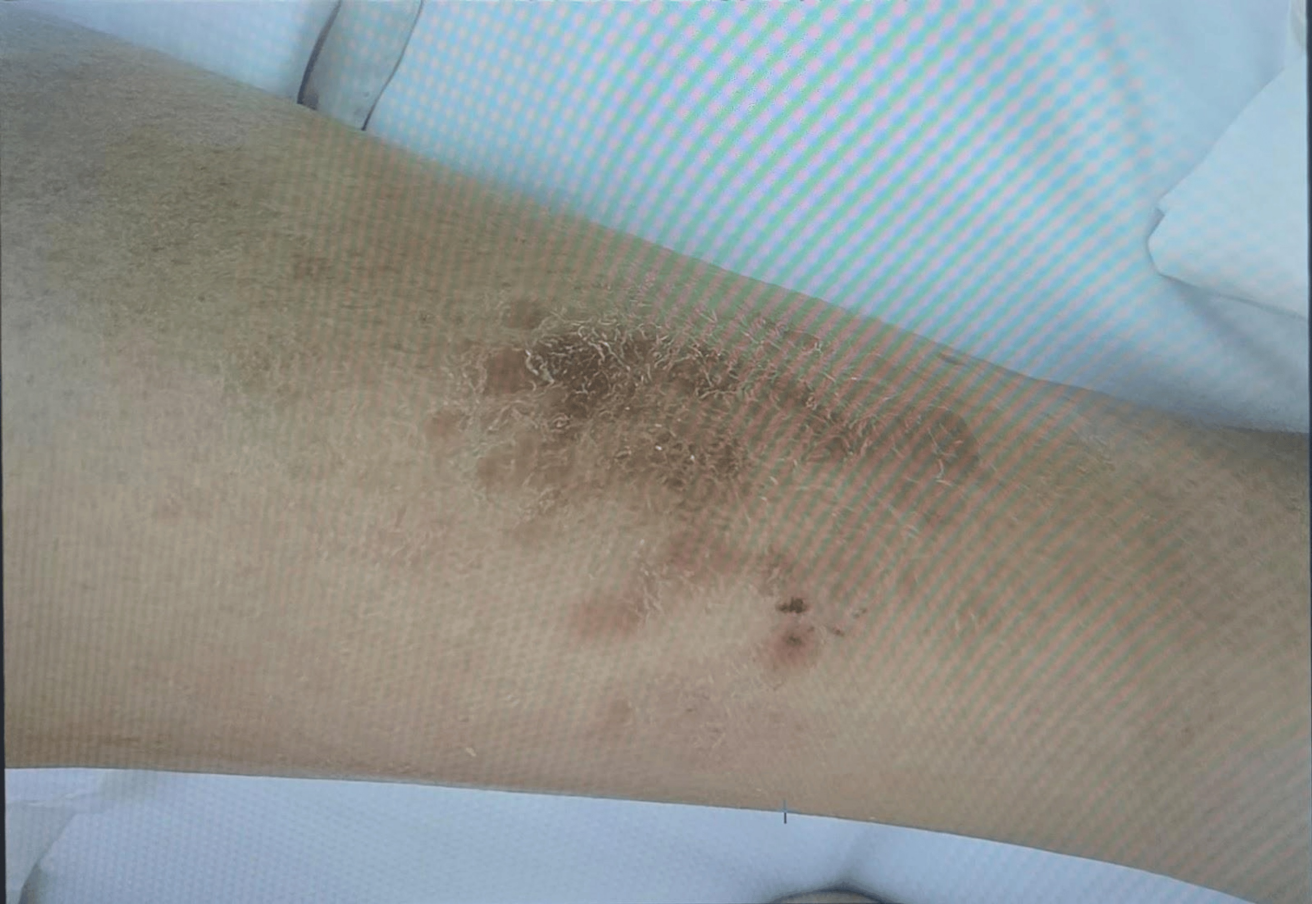
Petechial rash on left lower limb

Petechial rashes were observed over the lower back and bilateral lower limbs (Figures 2-4). There was no lower-limb swelling, peripheral pulses were intact, and neurological examination was unremarkable. 

Laboratory investigations

Initial laboratory testing demonstrated a mildly elevated D-dimer level. Haematological evaluation revealed transient leukocytosis that normalised within 24 hours and acute anaemia developing over four days, likely due to fragmentation haemolysis of RBCs by the fat emboli, while platelet counts remained within the normal range throughout admission. C-reactive protein levels increased on the second day of hospitalisation and gradually improved thereafter, explained by the activation of inflammatory cytokines due to free fatty acids in circulation (Table 1).

**Table 1:** Laboratory investigations showing acute anemia, raised CRP, and moderately raised D-dimer

Laboratory variables	Day 1 of admission	Day 2 of admission	Day 4 of admission	Reference Range
White blood cell count	12.9	4.2	6.6	3.6 - 11.0 x 10^3/UL
Hemoglobin	10.0	8.4	7.6	12 - 15 gm/dL
Red blood cell count	3.98	3.22	2.92	3.80 - 4.80 x 10^6/uL
Platelet count	317	249	320	150 - 410 x 10^3/uL
C-reactive protein	1.5	61.8	23.8	<5.0 mg/L
D-dimer	1.10	-	-	<0.5 ug/ml FEU

Imaging

**Figure 5:**
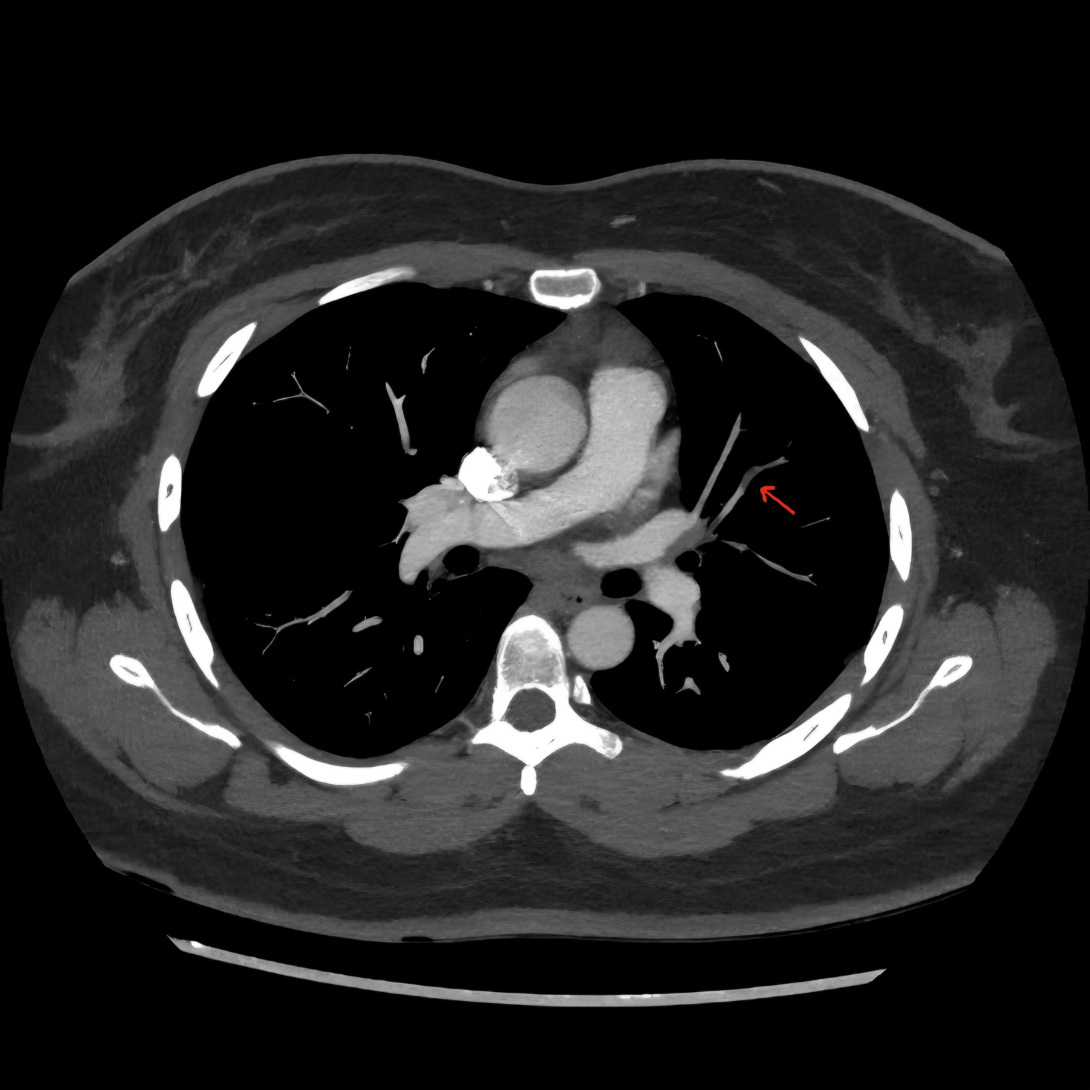
Axial CT pulmonary angiography showing a subtle intraluminal filling defect in a left lobe sub-segmental pulmonary artery. The arrow points to a very subtle, low-attenuation, and hypodense intraluminal filling defect in a left lobe sub-segmental pulmonary artery. The hypodensity is maximum at the genu of the pulmonary artery, most likely attributed to a mild fat embolism.

Computed tomography pulmonary angiography (CTPA) showed a subtle intraluminal filling defect in the left lobe sub-segmental pulmonary arteries, supporting the diagnosis of mild acute pulmonary embolism. However, no fat attenuation was done to the CT scan to differentiate fat embolism from thromboembolism (Figure 5). Doppler ultrasonography of the lower limbs demonstrated no evidence of deep vein thrombosis. A 12-lead ECG demonstrated a normal sinus rhythm with sinus tachycardia and no ischaemic changes.

Differential diagnoses

Fat embolism syndrome (FES) poses a diagnostic challenge due to significant overlap with several cardiopulmonary and neurological disorders. In particular, it must be differentiated from pulmonary thromboembolism, as well as other pulmonary conditions such as pulmonary contusion and pulmonary oedema, aspiration pneumonia, thrombotic thrombocytopenic purpura, immune thrombocytopenic purpura, blood dyscrasias such as leukemias, meningitis, encephalitis, and intracranial haemorrhage [9]. Local anaesthesia systemic toxicity (LAST) is another differential to be considered in this case. However, due to a lack of an operative procedure note, a lack of knowledge about the local anaesthetic dose given before liposuction, absence of symptoms of LAST on presentation, and confirmation of mild FES on CTPA, the treating physician excluded it from the list of differentials. 

Pulmonary thromboembolism (PE) typically presents with tachycardia, dyspnea, chest pain, hypoxemia, and elevated D-dimer levels, with symptoms developing within hours to days following a pro-thrombotic event. Unlike FES, PE does not account for petechial rash or the abrupt onset of neurological manifestations that are characteristic of fat embolism syndrome. CTPA with fat attenuation can differentiate between FES, pulmonary thromboembolism, and other lung pathologies [9].

The short interval between the procedure and onset of symptoms, absence of significant respiratory compromise, and presence of early haemodynamic symptoms are atypical for venous thromboembolism. In contrast, fat embolisation may occur immediately following adipose tissue disruption, supporting fat embolism as the most plausible mechanism [5, 7]. Fat emboli usually produce more subtle symptoms and are often clinically silent or present with vague, easily overlooked signs [6, 8].

Management and outcome

The patient was admitted for close medical monitoring. Oxygen saturation remained normal, with no respiratory compromise developing over the entire course of the hospital stay, supporting the possibility of a mild or atypical pulmonary fat embolism. 

Management was mainly supportive, including careful intravenous fluid administration, empirical antibiotic coverage (ceftriaxone) for raised inflammatory markers, therapeutic anticoagulation (enoxaparin) based on CTPA findings, and anti-arrhythmic therapy (bisoprolol 2.5 mg, as needed) for episodes of sinus tachycardia. No specific pharmacological treatment for fat embolism syndrome was initiated. Complete blood counts were ordered daily, and enoxaparin and ceftriaxone were discontinued four days later. The patient remained haemodynamically stable, demonstrated gradual clinical improvement with supportive care alone, and was discharged after four days of inpatient care.

## Discussion

Fat embolism syndrome is an uncommon but potentially life-threatening condition, commonly associated with long-bone fractures. However, it has also been reported in non-traumatic settings, including soft-tissue injury and elective cosmetic procedures such as liposuction [10]. Most of the reported causes of FES are summarised in Table 2 [4].

**Table 2:** Traumatic and non-traumatic causes of fat embolism syndrome Source: Reproduced from Uransilp et al. [4] under the CC BY 4.0 license.

Trauma related	Non-trauma related
1. Long bone fracture	1. Pancreatitis
2. Pelvic fracture	2. Diabetes mellitus
3. Fracture of other marrow-containing bones	3. Osteomyelitis and panniculitis
4. Orthopedic procedures	4. Bone tumour lysis
5. Soft tissue injuries (e.g., chest compression with or without rib fracture)	5. Steroid therapy
6. Burns	6. Sickle cell hemoglobinopathies
7. Liposuction, lipoinjection, fat grafting	7. Alcoholic liver disease
8. Bone marrow harvesting and transplant	8. Lipid infusion

Reviews of liposuction‑related FES describe a classical triad of respiratory distress, neurological impairment, and petechial rash, but highlight that many patients do not manifest all components and that diagnosis remains clinical, with nonspecific labs and imaging [11].

The pathogenesis of fat embolism syndrome is most commonly explained by the mechanical theory, which proposes that fat droplets enter the systemic circulation and cause mechanical obstruction of blood vessels, particularly within the pulmonary and cerebral micro-vasculature, which results in impaired perfusion, tissue ischaemia, and organ dysfunction [6]. Liposuction inherently disrupts adipose tissue and facilitates mobilisation of fat, thereby increasing the risk of fat embolisation [12].

The biochemical theory suggests that circulating fat globules undergo enzymatic degradation into free fatty acids and glycerol. These metabolites exert cytotoxic effects on vascular endothelium, triggering inflammatory cascades, increasing capillary permeability, promoting tissue oedema and haemorrhage, and multi-organ dysfunction, including respiratory dysfunction ranging from hypoxia to ARDS, neurological deterioration, and microvascular injury leading to petechial rash, retinal haemorrhages, anaemia, and thrombocytopenia [13, 14]. 

Fat Embolism Syndrome remains a clinical diagnosis of exclusion, after ruling out other causes of cardio-pulmonary or neurological deterioration [8]. Several scoring systems have been developed to aid in the diagnosis of fat embolism syndrome. 

The Gurd and Wilson criteria establish a diagnosis of FES based on a combination of major and minor clinical features. Major criteria include respiratory insufficiency (hypoxemia), neurological involvement (confusion), and a characteristic petechial rash, while minor criteria encompass systemic signs such as pyrexia, tachycardia, jaundice, and various hematological changes like anemia and thrombocytopenia. A diagnosis is traditionally supported by the presence of at least one major criterion plus four minor criteria, or the presence of two major criteria [2, 15].

The Fat Embolism Syndrome Index, described by Schonfeld et al., employs a quantitative scoring system where a cumulative score of more than five supports the diagnosis of FES. In this model, high point values are assigned to pathognomonic or severe signs, such as diffuse petechiae (5 points), alveolar infiltrates on chest radiographs (4 points), and hypoxemia (3 points). One point each is assigned for non-specific clinical markers, including confusion, fever, tachycardia, and tachypnea [16].

In our case, the clinical picture was consistent with an atypical form of fat embolism syndrome, presenting with tachycardia, dizziness, anaemia, and petechial rashes, fulfilling only two minor criteria of anaemia and tachycardia and one major criterion of petechial rash. Several case studies reported the development of symptoms within 2-24 hours of procedures such as liposuction and fracture-related FES, with earlier onset associated with a more severe course [5, 7]. In contrast, our case presented relatively early with a milder form of atypical FES.

There is no established threshold volume of aspirated fat associated with the development of fat embolism syndrome. Published cases demonstrate that FES may occur even after low- to moderate-volume liposuction, suggesting that technique, tissue trauma, and early intravascular fat entry are more critical determinants than total aspirate volume [5, 7, 12].

Across reviews and case series, there is consensus for a lack of specific pharmacologic therapy for FES; management is primarily supportive (oxygen/ventilatory support, haemodynamic stabilisation, ICU monitoring) [7, 11]. Corticosteroids have a controversial prophylactic and therapeutic role in FES, and in a haemodynamically stable, non-hypoxic patient such as ours, conservative management without steroids was considered reasonable [6, 8, 16]. Anticoagulation is not an established treatment for FES, but it can be used for standard postoperative venous thromboembolism prophylaxis, as in our case [8]. Several reports stress the importance of early recognition and aggressive supportive care to reduce mortality [5, 7, 11].

Strengths and limitations

A major strength of this case is the prompt diagnostic evaluation, including early CT pulmonary angiography and laboratory testing, which facilitated exclusion of alternative life-threatening diagnoses and timely supportive management.

Limitations include the absence of a detailed operative report and the atypical clinical presentation, which did not fulfill established diagnostic criteria for fat embolism syndrome. Other limitations include a lack of testing for fat macroglobulinemia, ESR, and fat attenuation on CTPA. Workup for acute anaemia was limited by the patient’s decision to decline additional testing. These factors contributed to diagnostic uncertainty and limited definitive confirmation of the diagnosis. Therefore, the presentation and investigations strongly support the diagnosis of an atypical fat embolism syndrome, but do not confirm the diagnosis. 

## Conclusions

This case describes an unusually early and atypical presentation of suspected fat embolism syndrome following cosmetic liposuction in a young, previously healthy woman. The patient presented with tachycardia, hypotension, petechial rashes, and ecchymosis one hour after undergoing elective cosmetic liposuction of the abdomen, while laboratory investigations revealed elevated D-dimer levels, CRP, and anemia. CTPA showed evidence of mild acute pulmonary embolism, most likely FES, based on the clinical criteria of FES partially fulfilled and exclusion of other differential diagnoses. Hence, FES was a clinical diagnosis of exclusion, which highlights the limitations of relying solely on classical diagnostic criteria and the importance of applying clinical reasoning when evaluating acute postoperative complications, even in the absence of classical features.

As the volume of cosmetic fat-mobilising procedures continues to increase, clinicians should remain alert to subtle and non-traditional presentations of fat embolism to ensure timely assessment and optimal patient safety. Vigilant monitoring and early diagnostic workup are crucial for ensuring patient safety following surgical procedures, especially fat-mobilising cosmetic interventions.
